# Shared and Cell-Type-Specific Gene Expression Patterns Associated With Autism Revealed by Integrative Regularized Non-Negative Matrix Factorization

**DOI:** 10.3389/fgene.2022.865371

**Published:** 2022-05-11

**Authors:** Jinting Guan, Yan Zhuang, Yue Kang, Guoli Ji

**Affiliations:** ^1^ Department of Automation, Xiamen University, Xiamen, China; ^2^ National Institute for Data Science in Health and Medicine, Xiamen University, Xiamen, China

**Keywords:** ASD, cell-type-specific gene module, shared gene module, gene function, integrative regularized non-negative matrix factorization

## Abstract

Human brain-related disorders, such as autism spectrum disorder (ASD), are often characterized by cell heterogeneity, as the cell atlas of brains consists of diverse cell types. There are commonality and specificity in gene expression among different cell types of brains; hence, there may also be commonality and specificity in dysregulated gene expression affected by ASD among brain cells. Moreover, as genes interact together, it is important to identify shared and cell-type-specific ASD-related gene modules for studying the cell heterogeneity of ASD. To this end, we propose integrative regularized non-negative matrix factorization (iRNMF) by imposing a new regularization based on integrative non-negative matrix factorization. Using iRNMF, we analyze gene expression data of multiple cell types of the human brain to obtain shared and cell-type-specific gene modules. Based on ASD risk genes, we identify shared and cell-type-specific ASD-associated gene modules. By analyzing these gene modules, we study the commonality and specificity among different cell types in dysregulated gene expression affected by ASD. The shared ASD-associated gene modules are mostly relevant to the functioning of synapses, while in different cell types, different kinds of gene functions may be specifically dysregulated in ASD, such as inhibitory extracellular ligand-gated ion channel activity in GABAergic interneurons and excitatory postsynaptic potential and ionotropic glutamate receptor signaling pathway in glutamatergic neurons. Our results provide new insights into the molecular mechanism and pathogenesis of ASD. The identification of shared and cell-type-specific ASD-related gene modules can facilitate the development of more targeted biomarkers and treatments for ASD.

## Introduction

The human brain is a highly heterogeneous organ, consisting of multiple kinds of cell types. Brain-related disorders, such as autism spectrum disorder (ASD), are often characterized by cell heterogeneity and mainly affect some specific cell types. ASD, a set of neuropsychiatric disorders, is characterized by highly genetic and phenotypic heterogeneity. To date, its actual causes and underlying mechanisms remain unclear. Although there have been hundreds of genes identified to be associated with ASD, they only account for 10–20% of ASD cases (Rylaarsdam and Guemez-Gamboa, 2019). Genes do not act alone, and what determines the manifestation of a disease in different cell types is the presence of disease-associated gene modules instead of individual genes (Kitsak et al., 2016; Guan et al., 2021). Moreover, as there are commonality and specificity in gene expression among different cell types of brains, there may also be commonality and specificity in dysregulated gene expression affected by ASD among brain cells. Therefore, based on gene expression datasets of multiple human brain cells, the detection of shared and cell-type-specific ASD-associated gene modules is of significance to study the molecular mechanism and pathogenesis of ASD.

Non-negative matrix factorization (NMF)-based methods have been developed and applied to the analyses of biological sequencing data, such as sparse NMF (sNMF) (Mairal et al., 2010) and sparse modular activity factorization (SMAF) (Cleary et al., 2017). In the context of integrating heterogeneous datasets, several methods have been proposed recently. Many of them were developed to integrate multi-modal or multi-omics data and focus on the analysis of samples, such as the joint definition of cell types of samples by taking the advantage of multiple heterogeneous datasets. For example, LIGER (Welch et al., 2019) was developed based on integrative non-negative matrix factorization (iNMF) (Yang and Michailidis, 2016) to factorize multiple datasets into a common gene-factor matrix, multiple dataset-specific gene-factor matrices, and multiple dataset-specific sample-factor matrices. Compared with the original algorithm of iNMF, LIGER adopted a novel block coordinate descent algorithm for performing iNMF, which can converge quickly. iNMF can extract consistent patterns embedded in various data sources by separating the homogeneous and heterogeneous effects among the sources, and it was mainly adopted to analyze the low-dimensional sample-factor matrices based on different kinds of data. The low-dimensional gene-factor matrices should be given more attention. The sparsity of sample representation (Yang and Michailidis, 2016) is beneficial to sample analyses, such as cell-type definition, while to perform gene module analyses, the sparsity or regularization of gene representation could be induced. Except for integrating multi-modal data, performing integrative and comparative analyses on the same type of data from multiple biological conditions, such as various cancer types or subtypes, various cell lines, and various cell types, is also valuable (Zhang and Zhang, 2019).

To depict the common and dataset-specific gene expression patterns, we proposed integrative regularized non-negative matrix factorization (iRNMF), by adopting iNMF and imposing a new regularization, to obtain a common gene-factor matrix and multiple dataset-specific gene-factor matrices. With iRNMF, we analyzed the gene expression data of multiple human brain cell types and obtained shared and cell-type-specific gene modules. Then, ASD-related risk genes were used to identify shared and cell-type-specific ASD-associated gene modules. By analyzing these gene modules, we studied the shared and cell-type-specific dysregulated gene expression patterns in ASD.

## Materials and Methods

### Integrative Regularized Non-Negative Matrix Factorization

Non-negative matrix factorization can factorize a high-dimensional gene expression matrix into two low-dimensional matrices, i.e., a gene-factor matrix and a sample-factor matrix, achieving the purpose of dimension reduction. To integrate and factorize multiple gene expression datasets into a common gene-factor matrix, multiple dataset-specific gene-factor matrices, and sample-factor matrices, iNMF (Yang and Michailidis, 2016) was proposed. The optimization problem is:

$$\underset{W,V_{1},\ldots ,V_{k},H_{1},\ldots H_{k}}{\text{min}}\overset{k}{\underset{i=1}{\sum }}\|X_{i}-H_{i}\left(W+V_{i}\right){\|}_{F}^{2}+\lambda \overset{k}{\underset{i=1}{\sum }}\|H_{i}V_{i}{\|}_{F}^{2},$$

$$\text{s}\text{.t}\text{.}\;\;W\geq 0,V_{i}\geq 0,H_{i}\geq 0,\;i=1,2,\ldots k,$$

where $X_{i}\in R^{n_{i}\times g}$ denotes each gene expression dataset, *g* denotes the number of genes, and $n_{i}$ denotes the number of samples in the *ith* dataset. $X_{i}$ is factorized into three low-dimensional matrices, $H_{i}\in R^{n_{i}\times m},W\in R^{m\times g},$ and $V_{i}\in R^{m\times g}$ , where *m* denotes the number of factors/gene modules. $H_{i}$ is the representation of samples in the low-dimensional space. $V_{i}$ and W are the dataset-specific and shared gene modules, respectively. λ is a regularization parameter.

The regularization of iNMF can make $V_{i}$ sparser to some degree, while to facilitate the analyses of shared and dataset-specific gene modules, we propose integrative regularized non-negative matrix factorization (iRNMF) by imposing a new regularization. The optimization problem is:

$$\underset{W,V_{1},\ldots ,V_{k},H_{1},\ldots H_{k}}{\text{min}}\overset{k}{\underset{i=1}{\sum }}\|X_{i}-H_{i}\left(W+V_{i}\right){\|}_{F}^{2}+\lambda_{\;1}\overset{k}{\underset{i=1}{\sum }}\|H_{i}V_{i}{\|}_{F}^{2}+\lambda_{2}\overset{k}{\underset{i=1}{\sum }}\|H_{i}W{\|}_{F}^{2},$$

$$\text{s}\text{.t}\text{.}\;\;W\geq 0,V_{i}\geq 0,H_{i}\geq 0,\;i=1,2,\ldots k,$$

where $\lambda_{\;1}$ and $\lambda_{2}$ are regularization parameters. The multiplicative updates often used for NMF-like optimization problems do not have a convergence guarantee and may need more iterations; therefore, we applied the block coordinate descent algorithm used in LIGER (Welch et al., 2019). We divided the variables into 2*k* + 1 blocks (corresponding to $H_{i}$ , $V_{i}$ for each dataset, and *W*) and performed block coordinate descent, iteratively minimizing the objective with respect to each block, holding the others fixed. We iterated:

$$W=\underset{W\geq 0}{argmin}||\left(\begin{array}{c} H_{1}\\ \vdots \\ H_{k}\\ \begin{array}{c} \sqrt{\lambda_{2}}H_{1}\\ \vdots \\ \sqrt{\lambda_{2}}H_{k} \end{array} \end{array}\right)W-{\left(\begin{array}{c} X_{1}-H_{1}V_{1}\\ \vdots \\ X_{k}-H_{k}V_{k}\\ O_{n_{1}\times g}\\ \vdots \\ O_{n_{k}\times g} \end{array}\right)}_{F}^{2}||,$$

$$H_{i}=\underset{H_{i}\geq 0}{argmin}||\left(\begin{array}{c} W^{T}+V_{i}^{T}\\ \sqrt{\lambda_{1}}V_{i}^{T}\\ \sqrt{\lambda_{2}}W^{T} \end{array}\right)H_{i}^{T}-{\left(\begin{array}{c} X_{i}^{T}\\ O_{g\times n_{i}}\\ O_{g\times n_{i}} \end{array}\right)}_{F}^{2}||,$$

$$V_{i}=\underset{V_{i}\geq 0}{argmin}||\left(\begin{array}{c} H_{i}\\ \sqrt{\lambda_{1}}H_{i} \end{array}\right)V_{i}-{\left(\begin{array}{c} X_{i}-H_{i}W\\ O_{n_{i}\times g} \end{array}\right)}_{F}^{2}||,$$

until convergence. Each of the optimization subproblems mentioned previously requires solving a non-negative least-squares problem, and we used the fast block principal pivoting algorithm (Kim et al., 2014) to solve each of these subproblems.

### Gene Expression Data

We downloaded the single-nucleus gene expression data derived from the middle temporal gyrus (MTG) of the human cortex (Hodge et al., 2019) from the Allen Institute for Brain Science. It includes 15,928 nuclei sampled from eight human donor brains, of which 15,206 were from postmortem donors with no known neuropsychiatric or neurological conditions and 722 were from distal and normal tissues of neurosurgical donors. We preprocessed the data with R packages of scatter (McCarthy et al., 2017) and scran (Lun et al., 2016), including the quality control of nuclei and genes, and removing a minority of nuclei assigned to different cell cycle phases by the function of cyclone in scran. Nuclear and mitochondrial genes downloaded from Human MitoCarta2.0 (Calvo et al., 2016) were excluded, and protein-coding genes were retained. After removing the nuclei not assigned to any specific cell type, we obtained the expression level of 17,120 protein-coding genes in 12,246 nuclei. Then, we used scran to obtain 7,011 highly variable protein-coding genes across all nuclei, which were defined as genes with biological components that are significantly greater than zero at a false discovery rate (FDR) of 0.1. After removing the cell types containing less than 20 nuclei, we obtained the gene expression data of nuclei from glutamatergic neuron (Gluta), GABAergic interneuron (GABA), astrocyte (Ast), oligodendrocyte (Oli), and oligodendrocyte precursor cell (OPC), including 8994, 2762, 227, 112, and 133 nuclei, respectively. The gene expression of 7,011 highly variable protein-coding genes in these five cell types was used for analyses.

### Determination of Parameters

To determine the number of factors/gene modules *m*, we used the same way with LIGER, applying Kullback–Leibler (KL) divergence as a criterion. When the number of factors is too low, factors will include many genes and samples will load on many factors, with the distribution of factor loadings for a particular sample approaching a uniform distribution (Welch et al., 2019). As the number of factors approaches the true number of gene modules, each sample will generally load on only a few factors. Therefore, we calculated the KL divergence, compared to a uniform distribution, of the factor loadings for each sample and plotted the median across samples as a function of *m* to select the saturation point of the curve as the optimal *m*. We also considered the mean squared error (MSE) between $X_{i}$ and the reconstructed data $\hat{X_{i}}$ , i.e., $\overset{k}{\underset{i=1}{\sum }}1/(\mathrm{ni}\times g)||X_{i}-H_{i}\left(W+V_{i}\right)|{|}_{F}^{2}$ , to help to determine the optimal *m*. To select the regularization parameters $\lambda_{\;1}\;$ and $\lambda_{2}$ , we applied the alignment metric (Butler et al., 2018) as a criterion, which LIGER also used, and plotted the alignment metric as a function of a combination of $\lambda_{\;1}$ and $\lambda_{2}$ to choose the point at which the alignment metric reaches the minimum value. $\lambda_{\;1}\;$ and $\lambda_{2}$ can be a value among 0.01, 0.1, 1, 10, and 100.

### Gene Module Analyses

We used iRNMF to analyze gene expression datasets of multiple cell types derived from human MTG. After obtaining the cell-type-specific and shared gene module matrices $V_{i}\;$ and  W , for each gene module, we calculated the z-scores of genes, and the genes whose z-scores are larger than one were regarded as module genes. The modules with no less than 20 module genes were reported. The gene modules significantly enriched with ASD genes were regarded as ASD-associated gene modules. ASD candidate genes were downloaded from the Simons Foundation Autism Research Initiative (SFARI), version of 2 September 2021. We identified ASD-associated gene modules by hypergeometric tests and performed the correction for multiple testing by the Bonferroni method (Rupert, 2012). Gene Ontology analysis was performed using the R package of clusterProfiler (Yu et al., 2012), with background genes set at the genes in the analyzed expression matrix. The GO term whose FDR-adjusted p-value < 0.1 and the number of genes in the term is not less than ten was reported.

## Results

### Overall Analytical Procedure

We proposed integrative regularized non-negative matrix factorization (iRNMF) to learn homogeneous and heterogeneous gene expression patterns across multiple datasets. Single-nucleus gene expression datasets of multiple cell types of human MTG (Hodge et al., 2019) were analyzed using iRNMF, involving glutamatergic neuron (Gluta), GABAergic interneuron (GABA), astrocyte (Ast), oligodendrocyte (Oli), and oligodendrocyte precursor cell (OPC) denoted by $X_{1},\ldots ,\;X_{5}$ , $X_{i}\in R^{n_{i}\times g}$ ,  i=1,2,…,5 , where *g* denotes the number of genes and $n_{i}$ denotes the number of samples in the *i*th cell type. iRNMF decomposed each gene expression dataset, $X_{i}$ , into three low-dimensional matrices, including the representation of samples in the low-dimensional space $H_{i}\in R^{n_{i}\times m}$ and the cell type-specific and shared gene module matrices $V_{i}\in R^{m\times g}$ and $W\in R^{m\times g}$ , respectively, where *m* denotes the number of factors/gene modules. As we study the shared and cell type-specific gene expression patterns across cells, we mainly focus on $V_{i}$ and W . Based on $V_{i}$ and W , for each gene module, we first calculated the z-scores of genes and determined the module genes as those with z-score > 1. The modules with no less than 20 module genes were reported. The gene modules determined from *W* were regarded as shared gene modules, and those determined from $V_{i}$ were regarded as cell-type-specific gene modules. Then, we identified the gene modules significantly enriched with SFARI ASD candidate genes using hypergeometric tests. The gene modules whose Bonferroni-adjusted hypergeometric test p-values < 0.1 were reported as ASD-associated gene modules. By analyzing the shared and cell-type-specific ASD-associated gene modules, we study the shared and cell-type-specific dysregulated gene expression across different cells in ASD.

### The Evaluation of Integrative Regularized Non-Negative Matrix Factorization

To show the effectiveness of iRNMF, we compared iRNMF with LIGER (which only imposes regularization on $\overset{k}{\underset{i=1}{\sum }}||H_{i}V_{i}|{|}_{F}^{2}$ but not on $\overset{k}{\underset{i=1}{\sum }}||H_{i}W|{|}_{F}^{2}$ ). First, we needed to determine the parameter values for LIGER and iRNMF. KL divergence was used to determine the optimal number of gene modules *m*, and the alignment metric (Butler et al., 2018) was used to determine the regularization parameters. For LIGER and iRNMF, we plotted the median of KL divergence across samples as a function of *m* to select the saturation point of the curve and also considered mean squared error (MSE), $\overset{k}{\underset{i=1}{\sum }}1/(\mathrm{ni}\times g)||X_{i}-H_{i}\left(W+V_{i}\right)|{|}_{F}^{2}$ (Supplementary Figure S1A). Thus, *m* was set to 100 for both LIGER and iRNMF. For LIGER, the regularization parameter λ was set to 1, which makes the alignment metric reach the minimum value (Supplementary Figure S1B). For iRNMF, we plotted the alignment metric as a function of a combination of $\lambda_{\;1}$ and $\lambda_{2}$ . The parameter values $\lambda_{\;1}$ = 0.01 and $\lambda_{2}$ = 10 can make the alignment metric reach the minimum value, while we noticed that $\lambda_{\;1}$ = 1 and $\lambda_{2}$ = 10 can give the second smallest alignment metric (Supplementary Figure S1C). The regularization parameter of LIGER was determined as 1, which is actually our $\lambda_{\;1}$ ; to better compare iRNMF with LIGER and analyze the effectiveness of the newly added constraint, we chose $\lambda_{\;1}$ = 1 and $\lambda_{2}$ = 10 instead. The reason why we chose the minimum alignment instead of the maximum alignment as a criterion is that the input datasets are from different cell types and they could not be aligned together. The alignment metric measures the uniformity of mixing for multiple samples in the aligned latent space, which should be high when datasets share underlying cell types and low when datasets do not share cognate populations (Butler et al., 2018; Welch et al., 2019). As our analyzed datasets are from different cell types, we used the minimum alignment to determine the regularization parameters.

Next, we compared iRNMF with LIGER based on cell representation. For each cell type, we calculated the Pearson correlation between flatten $X_{i}$ and $\hat{X_{i}}=H_{i}\left(W+V_{i}\right)$ (Supplementary Figure S2A) to evaluate the reconstruction. Also, we calculated sample–sample distance matrices using $X_{i}$ and $\hat{X_{i}}$ and then flatten the distance matrices to calculate their Pearson correlation (Supplementary Figure S2B). Both correlations of iRNMF are slightly better than those of LIGER. Then, for each cell, we calculated the Pearson correlation between the gene expression levels of this cell in $X_{i}$ and $\hat{X_{i}}$ (Supplementary Figure S2C). We found that iRNMF is better than LIGER in the cell type GABA, and in the other four cell types, iRNMF and LIGER are evenly matched. To compare the low-dimensional $H_{i}$ obtained from LIGER and iRNMF, we combined all $H_{i}$ as H and performed cell clustering based on H to check if different cell types are distinguishable. We performed K-means based on H and calculated the clustering indexes, including ARI (adjusted Rand index), FMI (Fowlkes and Mallows index), JC (Jaccard coefficient), NMI (normalized mutual information), PUR (purity), and SC (silhouette coefficient) (Supplementary Figure S2D). It can be noted that the clustering performances of iRNMF are better than those of LIGER when being faced with datasets of different cell types.

Lastly, we compared the gene modules obtained using LIGER and iRNMF. We calculated gene–gene correlation matrices using $X_{i}$ and $\hat{X_{i}}$ and then flatten the correlation matrices to calculate their Pearson correlation (Supplementary Figure S2E). We also calculated gene–gene correlation matrices using $X_{i}$ and low-dimensional gene representation $W+V_{i}$ and then calculated their Pearson correlation (Supplementary Figure S2F). It can be seen that both correlations of iRNMF are better than those of LIGER. Then, for each gene, we calculated the Pearson correlation between the expression levels of this gene in $X_{i}$ and $\hat{X_{i}}$ (Supplementary Figure S2G). The correlations of iRNMF are significantly higher than those of LIGER in four cell types, except for Gluta, in which iRNMF and LIGER are evenly matched. Moreover, we expected that different modules should represent distinct biological functions and should not overlap too much. To evaluate the distinct biological functions of gene modules, we adopted the evaluation way as in Cleary et al (2017), using the number of uniquely enriched gene sets. For each gene module of *W* and $V_{i}$ , we tested it for enrichment in GO terms and considered its top five significant GO terms. Then, we identified the uniquely enriched GO terms of each module, which are the terms enriched in at most one module of this considered cell type, and calculated the average number of unique gene sets per module (Supplementary Figure S2H). It can be seen that in all cell types, the number of uniquely enriched GO terms of iRNMF is larger than that of LIGER. The comparisons indicate that iRNMF is effective, and the obtained low-dimensional matrices are helpful for subsequent gene module analysis.

### Shared Gene Expression Patterns Associated With Autism Spectrum Disorder

**FIGURE 1:**
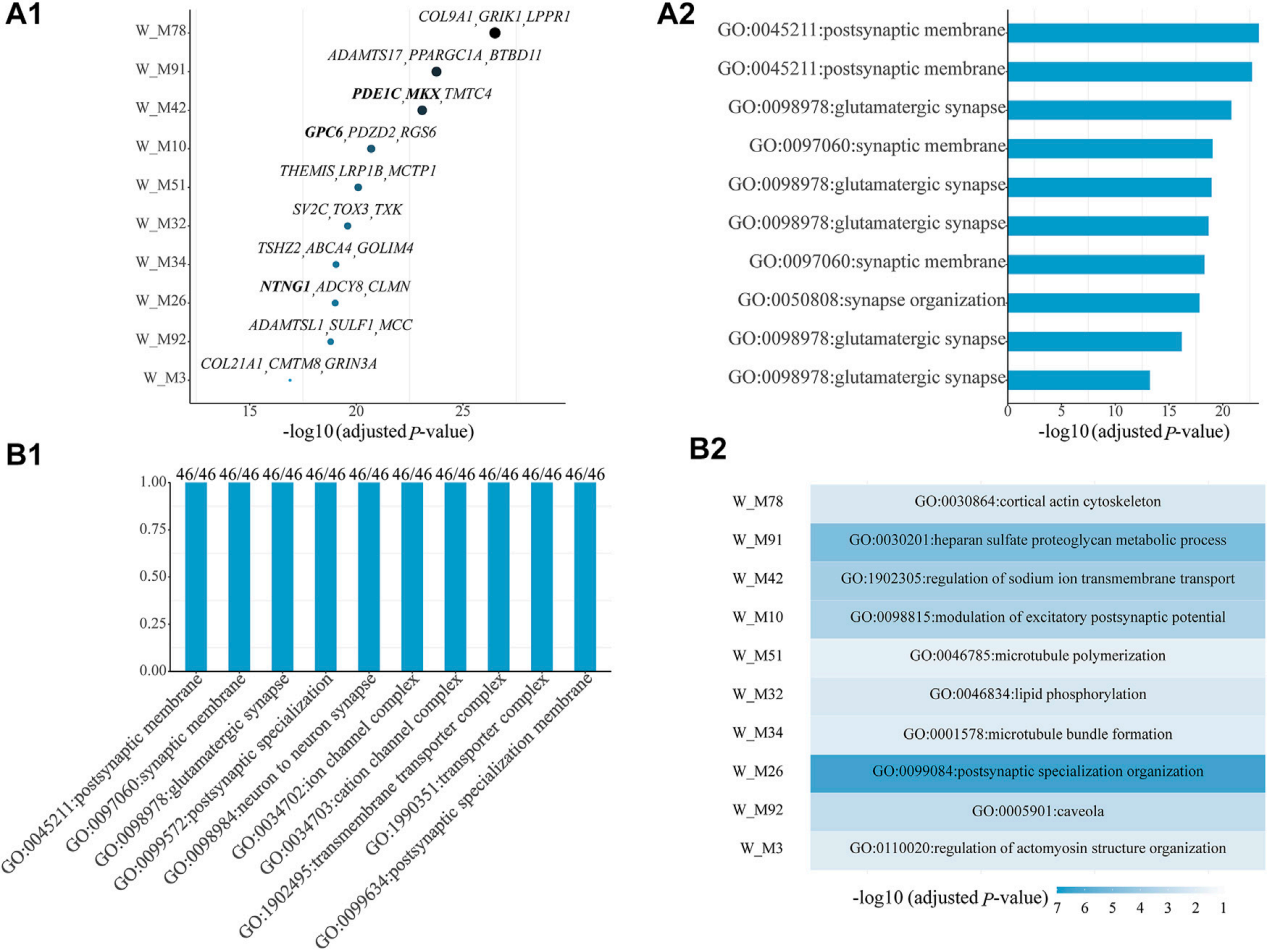
Top ten shared ASD-associated gene modules along with **(A1)** their Bonferroni-adjusted *p*-values, top three z-score genes, and **(A2)** top one significant GO term. **(B1)** Top ten common enriched GO terms among all shared ASD-associated gene modules along with the frequency of occurrence and the total number of gene modules. **(B2)** Module-specific top one GO term of the top ten shared ASD-associated modules. The SFARI ASD genes are bold. The Bonferroni-adjusted p-values were derived from the hypergeometric tests using module genes and ASD genes.

Among all shared gene modules determined from *W*, 46 are significantly enriched with ASD genes (Supplementary Table S1). For the top ten shared ASD-associated gene modules, we list their Bonferroni-adjusted p-values, top three z-score genes (Figure 1A1), and top one significant GO term (Figure 1A2). Some top genes are ASD genes, including *PDE1C* and *MKX* in W_M42, *GPC6* in W_M10, and *NTNG1* in W_M26. The top one significant GO term is all related to synapses, whose dysregulation has been proven to be associated with ASD. Then, we checked which kinds of GO terms are the most common among all GO terms of all shared ASD-associated gene modules and found that the top ten common GO terms are also associated with the functioning of synapses, appearing in all shared ASD-associated gene modules (Figure 1B1). Next, we focused on the modules which have module-specific gene functions, by removing the repeated GO terms between gene modules. There are 36 shared ASD-associated gene modules with module-specific GO terms (Supplementary Table S2). The top ten modules with module-specific gene functions are also the ones shown in Figure 1A1, and their top one module-specific GO term is shown in Figure 1B2. The top three modules most significantly enriched with ASD genes, W_M78, W_M91, and W_M42, are related to cortical actin cytoskeleton, heparan sulfate proteoglycan metabolic process, and regulation of sodium ion transmembrane transport, respectively. The actin cytoskeleton has been associated with ASD and provides a strategy for ASD treatment by targeting actin regulators (Duffney et al., 2015; Hlushchenko et al., 2018). The lacking of heparin sulfate, a proteoglycan involved in a variety of neurodevelopmental processes, has been correlated with ASD (Irie et al., 2012; Pérez et al., 2016). Ion channels, including sodium, calcium, and potassium, are implicated in the etiology of ASD (Daghsni et al., 2018). It can be seen that the identified gene modules are meaningful.

### Cell-Type-Specific Gene Expression Patterns Associated With Autism Spectrum Disorder

**FIGURE 2:**
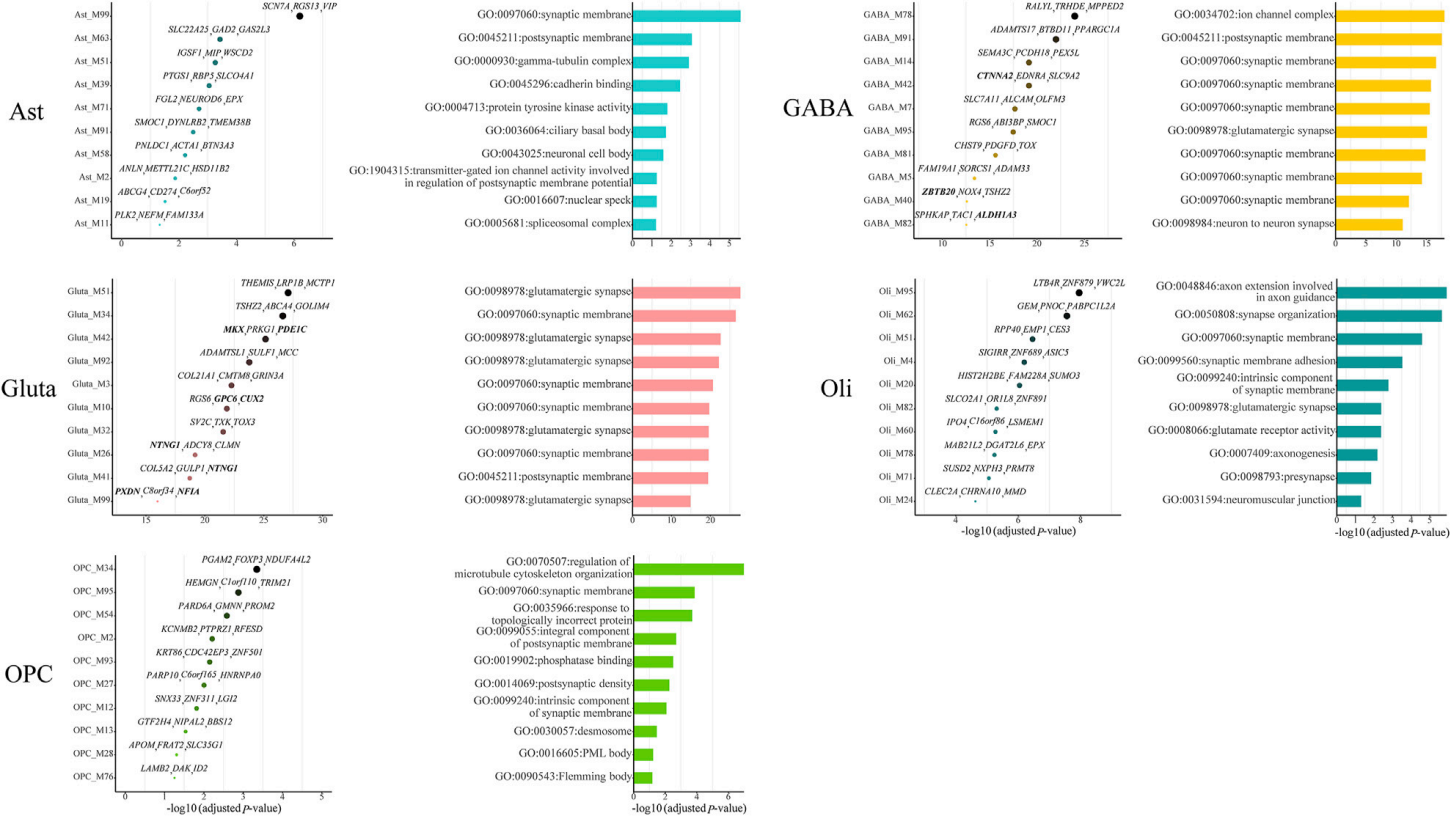
Top ten cell-type-specific ASD-associated gene modules along with their Bonferroni-adjusted hypergeometric test p-values, top three z-score genes, and top one significant GO term. The SFARI ASD genes are bold. The Bonferroni-adjusted p-values were derived from the hypergeometric tests using module genes and ASD genes.

Among all cell-type-specific gene modules, we identified 11, 25, 29, 45, and 14 cell-type-specific ASD-associated gene modules for Ast, GABA, Gluta, Oli, and OPC, respectively (Supplementary Table S1). We list the top ten significant gene modules along with their Bonferroni-adjusted p-values, top three z-score genes, and top one significant GO term (Figure 2). Noted that for the two kinds of neurons, GABA and Gluta, the cell-type-specific ASD-associated gene modules are more significantly enriched with ASD genes and more top three genes are ASD genes, compared with glial cells. Many of the top GO terms of cell-type-specific ASD-associated gene modules are related to synapses, while different gene functions may still be dysregulated in different cell types. For instance, gamma-tubulin complex, cadherin binding, and protein tyrosine kinase activity are associated with Ast-specific ASD-associated gene modules; regulation of microtubule cytoskeleton organization, phosphatase binding, and desmosome are significant in OPC-specific ASD-associated gene modules. These may indicate that different gene functions may be dysregulated by ASD in different cells, demonstrating the cell heterogeneity of ASD.

**FIGURE 3:**
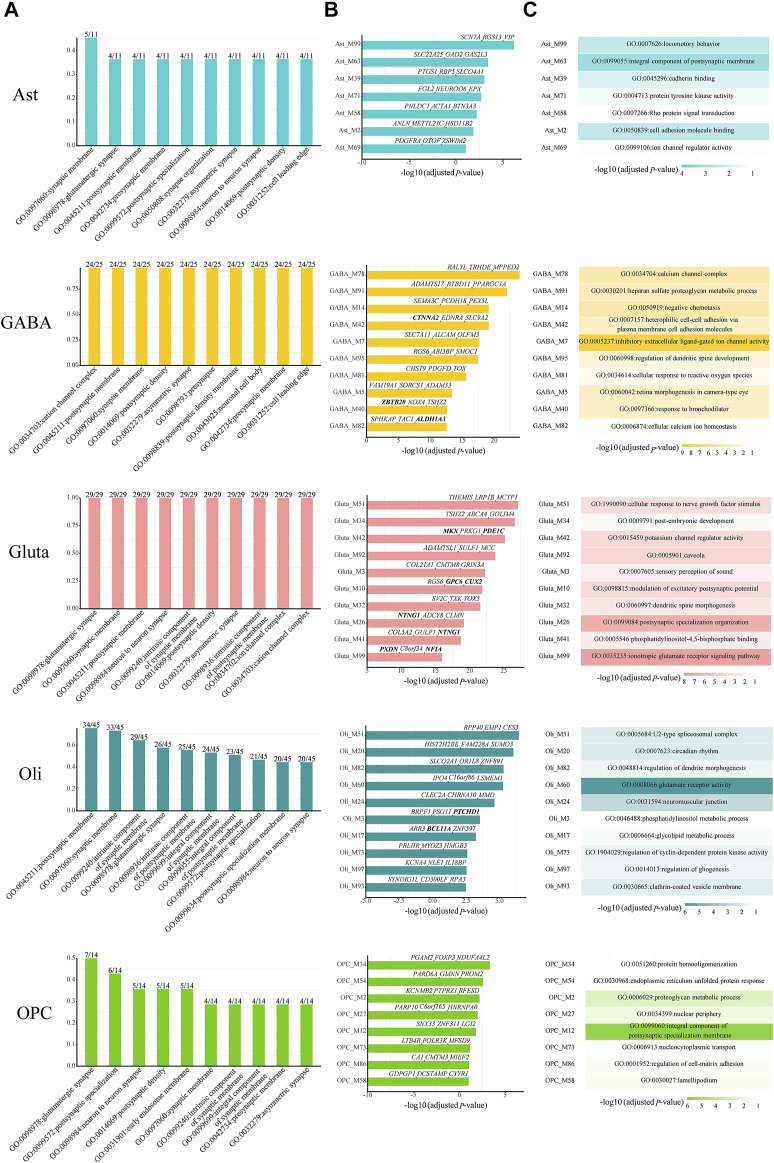
**(A)** Top ten common enriched GO terms among all cell-type-specific ASD-associated gene modules along with the frequency of occurrence and the total number of cell-type-specific ASD-associated gene modules. For the top ten cell-type-specific ASD-associated modules, which have module-specific GO terms, **(B)** their Bonferroni-adjusted hypergeometric test p-values, top three z-score genes, and **(C)** top one GO term are shown. The SFARI ASD genes are bold. The Bonferroni-adjusted p-values were derived from the hypergeometric tests using module genes and ASD genes.

Then, we checked which kinds of GO terms are the most common among all cell-type-specific ASD-associated gene modules in each cell type. Indeed, the functioning of synapses is important across all cell types (Figure 3A). Next, we focused on the modules which have module-specific gene functions. There are 7, 23, 24, 24, and 8 cell-type-specific ASD-associated gene modules left in Ast, GABA, Gluta, Oli, and OPC, respectively (Supplementary Table S2). We reported the top ten, along with their top three z-score genes (Figure 3B), and top one GO term (Figure 3C). In Ast, locomotory behavior, integral component of the postsynaptic membrane, and cadherin binding are functions specific to the top three modules, Ast_M99, Ast_M63, and Ast_M39. For GABA, it can be noted that inhibitory extracellular ligand-gated ion channel activity is specific to GABA_M7. On the contrary, the modulation of excitatory postsynaptic potential and ionotropic glutamate receptor signaling pathway are specific to Gluta_M10 and Gluta_M99, respectively. These gene functions are obviously associated with particular cell types. Neurons communicate with one another at synapses using two types of signals, electrical and chemical signals. At an electrical synapse, ions flow directly between cells. At a chemical synapse, neurotransmitters pass messages from the presynaptic to the postsynaptic neuron. The major excitatory and inhibitory neurotransmitters in brains are glutamate and GABA (gamma-aminobutyric acid), respectively. For Oli, regulation of dendrite morphogenesis and regulation of gliogenesis are specific to Oli_M82 and Oli_M97. For OPC, protein homooligomerization and endoplasmic reticulum unfolded protein response are specific to the top two modules, OPC_M34 and OPC_M54, respectively. The analysis of module-specific gene functions and top genes of cell-type-specific ASD-related gene modules can facilitate the development of more targeted biomarkers and treatments for ASD.

**FIGURE 4:**
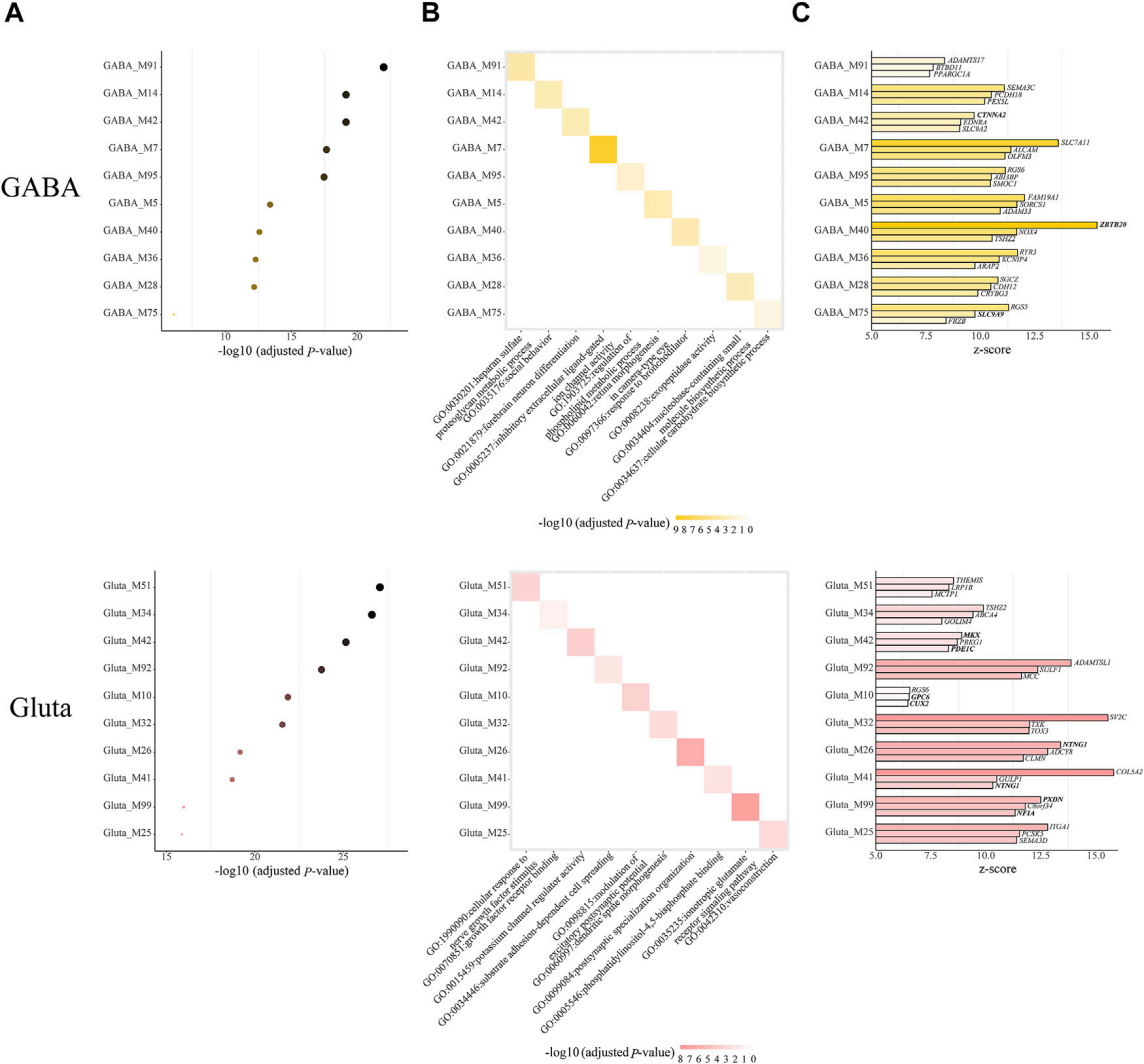
Cell-type-specific ASD-associated modules, which have both module-specific and cell type-specific GO terms, along with **(A)** their Bonferroni-adjusted hypergeometric test p-values, **(B)** top one enriched GO term, and **(C)** top three z-score genes. The SFARI ASD genes are bold. The Bonferroni-adjusted p-values were derived from the hypergeometric tests using module genes and ASD genes.

Next, we further examined the modules with both cell-type-specific and module-specific gene functions, which are those GO terms that only appear in one module of one cell type. In GABA, Gluta, Oli, and OPC, there are 14, 18, 1, and 1 cell-type-specific ASD-associated gene modules that have both cell-type-specific and module-specific gene functions (Supplementary Table S3). It can be noted that more modules have cell type-specific and module-specific gene functions in neuronal cells, emphasizing the neurons are mainly affected by ASD. For the cell types with more than one cell-type-specific ASD-associated gene modules, we show the top ten modules along with their Bonferroni-adjusted p-values, the enriched top one GO term, and the top three z-score genes (Figure 4). Among the top three genes, *CTNNA2* in GABA_M42, *ZBTB2* in GABA_M40, and *SLC9A9* in GABA_M75 are ASD genes. *MKX* and *PDE1C* in Gluta*_*M42, *GPC6* and *CUX2* in Gluta_M10, and *PXDN* and *NFIA* in Gluta_M99 are ASD genes. These gene modules may need more attention. We note that different kinds of gene functions are specific to ASD-associated modules of different cell types. GABA-specific ASD-associated gene modules are responsible for inhibitory extracellular ligand-gated ion channel activity and forebrain neuron differentiation, and so on. Gluta-specific ASD-associated gene modules are responsible for nerve growth factors, excitatory postsynaptic potential, and ionotropic glutamate receptor signaling pathway, and so on. Oli_M60 and OPC_M12 have a cell-type-specific and module-specific function, regulation of bone mineralization and lipid transporter activity, respectively (Supplementary Table S3). These results indicate that in different cell types, different kinds of gene functions may be specifically dysregulated in ASD, highlighting the cell heterogeneity of ASD.

## Discussion

Brain-related diseases are often characterized by cell heterogeneity and mainly affect some specific cell types, as the brain is highly heterogeneous. To study the common and cell type-specific gene expression patterns across different brain cell types, we proposed iRNMF by adopting iNMF and imposing a further regularization. With iRNMF, we analyzed the gene expression data of multiple human brain cell types to obtain shared and cell-type-specific gene modules and cell-type-specific cell representations. By comparing iRNMF with LIGER in terms of cell representations and gene modules, it has been shown that iRNMF is effective, and the obtained low-dimensional matrices are beneficial for the downstream analyses, especially gene module analyses.

By using curated ASD candidate genes, shared and cell-type-specific ASD-associated gene modules were identified. For the shared ASD-associated gene modules, their significant gene functions are mostly relevant to the functioning of synapses, which has already been proven to be associated with ASD. Then, we identified the module-specific gene functions, including cortical actin cytoskeleton, heparan sulfate proteoglycan metabolic process, and regulation of sodium ion transmembrane transport. As to cell-type-specific ASD-associated gene modules, GABA-specific and Gluta-specific ASD-associated gene modules are more significantly enriched with ASD genes, and more top three genes are ASD genes compared with glial cells, emphasizing that the neurons are mainly affected by ASD. Many top GO terms of cell-type-specific ASD-associated gene modules are related to synapses, while different gene functions may still be specifically dysregulated by ASD in different cell types. Therefore, we focused on the functions which are specific to modules and also cell types. We noted that inhibitory extracellular ligand-gated ion channel activity and forebrain neuron differentiation are functions specifically significant in GABA; nerve growth factor, excitatory postsynaptic potential, and ionotropic glutamate receptor signaling pathway are specifically related to Gluta; lipid transporter activity is specifically significant in OPC.

By analyzing the gene functions and top important genes of shared and cell-type-specific ASD-associated gene modules, we study the shared and cell-type-specific dysregulated gene expression patterns in ASD. Moreover, we highlighted the shared ASD-associated gene modules, which have module-specific gene functions, and cell-type-specific ASD-associated gene modules, which have both module-specific and cell-type-specific gene functions. Analyzing these gene modules can facilitate the development of more targeted biomarkers and treatments for ASD. Our results provide new insights into the molecular mechanism and pathogenesis of ASD, studying the cell heterogeneity of ASD. Our method can also be used to extract homogeneous and heterogeneous patterns embedded in data from multiple biological conditions, such as various cancer types or subtypes and various cell lines.

## Data Availability

The original contributions presented in the study are included in the article/Supplementary Material; further inquiries can be directed to the corresponding author.

## Supplementary Material

The Supplementary Material for this article can be found online at: https://www.frontiersin.org/articles/10.3389/fgene.2022.865371/full#supplementary-material

Supplementary Figure S1 Selection of parameter values of iRNMF and LIGER. **(A)** Selection of *m* using mean squared error (MSE) and KL divergence as criteria for iRNMF and LIGER. **(B)** Selection of the regularization parameter λ using alignment metric for LIGER. **(C)** Selection of the combination of regularization parameters ( $\lambda_{\;1}$ and $\lambda_{2}$ ) using alignment metric for iRNMF.

Supplementary Figure S2 Comparisons between iRNMF and LIGER. **(A)** Pearson correlation coefficient between original data $X_{i}$ and reconstructed data $\hat{X_{i}}$ . **(B)** Pearson correlation coefficient between sample–sample distance matrices calculated from $X_{i}$ and $\hat{X_{i}}$ . **(C)** Pearson correlation between the gene expression levels of each cell in $X_{i}$ and $\hat{X_{i}}$ . **(D)** Performances of clustering based on *H*, including ARI, FMI, JC, NMI, PUR, and SC. **(E)** Pearson correlation coefficient between gene–gene correlation matrices calculated from $X_{i}$ and $\hat{X_{i}}$ . **(F)** Pearson correlation coefficient between gene–gene correlation matrices calculated from $X_{i}$ and low-dimensional gene representation $W+V_{i}$ . **(G)** Pearson correlation between the expression levels of each gene in $X_{i}$ and $\hat{X_{i}}$ . **(H)** Number of uniquely enriched GO terms. ns denotes not significant; * denotes *p* < 0.05, ** denotes *p* < 0.01, *** denotes *p* < 0.001, and **** denotes *p* < 0.0001.

Supplementary Table S1 Shared and cell-type-specific ASD-associated gene modules. For these modules, their Bonferroni-adjusted hypergeometric *p*-values, module genes sorted by z-scores, and enriched gene functions are listed.

Supplementary Table S2 Shared and cell-type-specific ASD-associated gene modules that have module-specific gene functions. For these modules, their Bonferroni-adjusted hypergeometric *p*-values, module genes sorted by z-scores, and enriched module-specific gene functions are listed.

Supplementary Table S3 Cell-type-specific ASD-associated gene modules that have both module-specific and cell type-specific gene functions. For these modules, their Bonferroni-adjusted hypergeometric *p*-values, module genes sorted by z-scores, and enriched module-specific and cell-type-specific gene functions are listed.
