# Supplementary figures and images for: Shared and Cell-Type-Specific Gene Expression Patterns Associated With Autism Revealed by Integrative Regularized Non-Negative Matrix Factorization
Source: Front Genet. 2022 May 11;13:865371. doi: 10.3389/fgene.2022.865371 (PMC9130660; doi:10.3389/fgene.2022.865371)

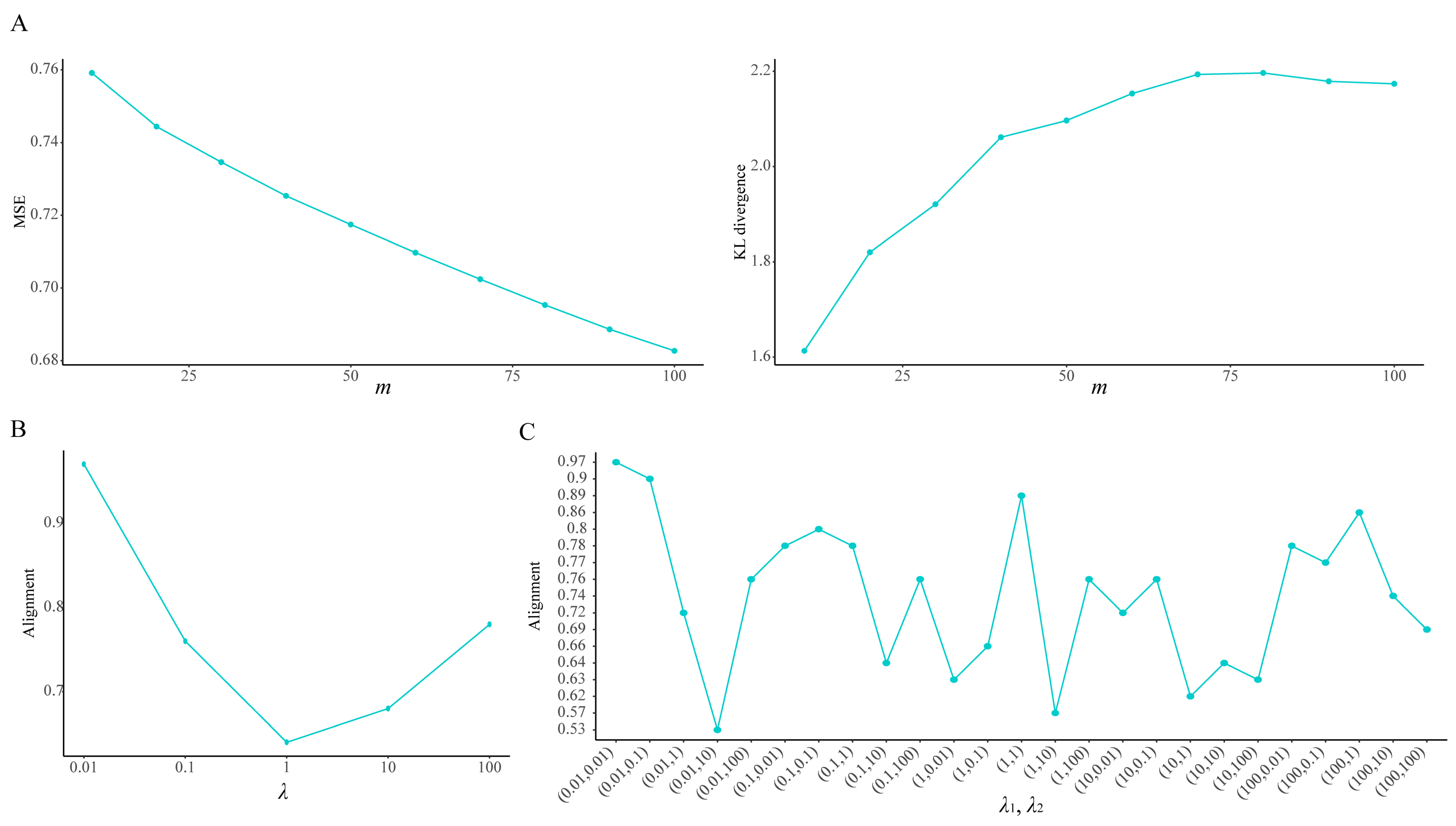

Supplement: Supplementary file 3 [file Image1.TIF]
